# Towards spontaneous parametric down conversion from monolayer MoS_2_

**DOI:** 10.1038/s41598-018-22270-4

**Published:** 2018-03-01

**Authors:** Hatef Dinparasti Saleh, Stefano Vezzoli, Lucia Caspani, Artur Branny, Santosh Kumar, Brian D. Gerardot, Daniele Faccio

**Affiliations:** 1 Institute of Photonics and Quantum Sciences, SUPA, Heriot-Watt University, Edinburgh, EH14 4AS United Kingdom; 2 Institute of Photonics, Department of Physics, University of Strathclyde, Glasgow, G1 1RD United Kingdom

**Keywords:** Nonlinear optics, Quantum optics, Sub-wavelength optics

## Abstract

We present a detailed study of the second order nonlinearity of 2D (mono-atomic layer) dichalcogenide MoS_2_, both in the visible and in the IR regime, and test its potential for spontaneous parametric down-conversion (SPDC), the amplification of vacuum fluctuations mediated by optical nonlinearity. We develop a model of SPDC from a deeply subwavelength nonlinear medium, where phase matching conditions are completely relaxed, and make predictions about the rate of emitted photons, their momentum, polarisation and spectrum. We show that detection in the visible spectral region is hindered by the strong photoluminescence background. Moving to the IR regime we observe indications of SPDC by performing polarization, power dependence and lifetime measurements around 1560 nm. We show that the signal from a single monolayer is qualitatively different from that generated by multi-layer MoS_2_. Finally, we characterize the latter as a new kind of photo-luminescence emission which is enhanced at the edges of multi-layer MoS_2_.

## Introduction

Since the discovery of graphene in 2004^1^, research on 2D materials has been growing at a high rate, motivated by huge potential for novel electronic and optoelectronic applications. Atomically thin layers of many different 2D materials ranging from wide-bandgap insulators and semiconductors to superconductors can be easily and inexpensively isolated from a piece of bulk, layered material^1^. Because of their intrinsic bandgap in the visible part of the spectrum which can be tuned with the number of layers, transition metal dichalcogenides (TMDC) are a family of 2D materials with largest potential for photonics. The most extensively studied member of this family is MoS_2_. When thinned down to a monolayer, MoS_2_ changes from an indirect bandgap semiconductor with an energy gap of *~1*.*29 eV* to a direct bandgap semiconductor with a bandgap of about 1.88 eV, due to the effect of quantum confinement on the material’s electronic structure. Because of this, 2D MoS_2_ shows an enhanced photoluminescence, as reported in recent seminal papers^2,3^. Many additional peculiar optical properties have been reported in 2D materials since huge exciton binding energy^1^, valley-polarized photoluminescence emission (when one valley is optically pumped with circularly polarized light)^4^, or the recent observation of single-photon emitters in WSe_2_ with very narrow emission linewidth (*~100* *μeV*) due to localized excitonic states^5–11^. Whilst most of these studies focused on linear optical properties, nonlinear optical effects are important aspects of light-matter interactions for relatively high excitation power and can play important roles in various photonic and optoelectronic applications. For instance, some groups investigated a possible application of MoS_2_ few-layers flakes as ultrafast and broadband saturable absorbers for laser Q-switching^12,13^.

A MoS_2_ crystal with 2 H stacking order has a layered structure with a single layer of Mo atoms sandwiched between two layers of S atoms in the *D*_*6ℎ*_ crystal symmetry, which is inversion symmetric (centrosymmetric). Hence, its second-order nonlinear response should vanish, and this is demonstrated by early experiments which measured a second-order nonlinear susceptibility ${\chi }^{(2)}$ of *2 H* bulk MoS_2_ of at most *0*.*01 pm/V*^14^. However, when thinned down to monolayer, the crystal structure of MoS_2_ reduces to *D*_*3ℎ*_, which has broken inversion symmetry. Because of the crystal symmetry changing with the number of layers, a strong second harmonic generation (SHG) has been observed in odd numbers of layers, in particular in monolayers^15–17^. The SHG signal from 2D MoS_2_ flakes is estimated to be a few orders of magnitude larger than that of common bulk materials such as LiNbO_3_ and β-BaB_2_O_4_ (BBO) (*7 to 9 pm/V*). However theoretical estimations and experimental values for the ${\chi }^{(2)}$ currently reported in literature are contrasting and vary by over 3 orders of magnitude. Some authors^18^ estimated a susceptibility of the order of *100 pm/V*, with on-resonance values rising to *4000 pm/V* around *800 nm*. This is 1–2 orders of magnitude less than the value of *10*^5^* pm/V* reported in^15^, while^16,17^ reported values about 1 order of magnitude inferior. CVD-grown or mechanically exfoliated samples can also lead to very different results: for instance^15^, reported values of ${\chi }^{(2)}$ at around 10^5^* pm/V* for exfoliated samples and *5 *×* 10*^3^* pm/V* for CVD (20 times less) for SHG measured at the fundamental pump wavelength of *810 nm*. In another work^19^, the authors investigate SHG by pumping in the IR region between *1100* and *2000 nm*. They found that CVD MoS_2_ flakes have values of ${\chi }^{(2)}$ around *400 pm/V*. The values found are 10 times smaller than the ones found by^15^ at 810 nm and comparable to the values found by^17^ and^16^ for exfoliated samples pumped around *810 nm*. This inconsistency of values could be due to the different methods used for the measurement. Notwithstanding these differences, all studies agree on the fact that monolayer MoS_2_ possesses a remarkably large second order nonlinearity.

This enhancement of is thought to be mainly due to excitonic resonances and one work suggested that MoS_2_ monolayer has an effective nonlinear susceptibility that is similar to that of absorbing nonlinear crystals such as GaAs and Te^17^. Some theoretical work^20^ demonstrated how electron-hole attraction significantly increases the magnitude of the SHG relative to the one-electron case. The spectral dependence (dispersion) of the second order nonlinearity both in the visible and near IR spectra is also not fully understood. Several theoretical papers^18–20^, have tried to interpret the experimental results using different models and showed that an excitonic theory can roughly reproduce the position of the peaks in the ${\chi }^{(2)}$ dispersion. Other groups also investigated the role of edge states and showed a resonant enhancement of SHG at the edges of MoS_2_ monolayers around *1310 nm*^21^.

Whilst most of the above-mentioned works on the second-order nonlinearity focussed on SHG as an optical imaging method for non-invasive determination of crystalline orientation and thickness, here we explore the possibility in MoS_2_ monolayers of another nonlinear process which relies on the second-order susceptibility, namely spontaneous parametric down-conversion (SPDC). SPDC in nonlinear crystals such as beta barium borate (BBO) or periodically poled potassium titanyl phosphate (PPKTP) are a workhorse in quantum optics for the generation of entangled photon pairs^22–24^. In SPDC one pump photon at high energy is converted into 2 twin photons of lower energy through the parametric amplification of vacuum fluctuations. SPDC is tightly linked to SHG: its efficiency is proportional to the second order nonlinear susceptibility and like SHG, requires careful phase matching conditions. However, in the low gain regime, the generation rate in SPDC scales linearly with the pump average power^25,26^. In principle, a monolayer of MoS_2_ of *0*.*62 nm* thickness could be as efficient as a *1 µm* BBO crystal in generating pairs of photons from the vacuum for given power (considering a *4000 pm/V* nonlinear susceptibility for monoloayer MoS_2_ and that the generation efficiency scales linearly with the crystal length in the low-gain regime). Efficient down conversion requires the phase mismatch term *ΔkL* to be close to zero, where *Δk* = *k*_*p*_ − *k*_*i*_ − *k*_*s*_, *k*_*p*_, *k*_*i*_, and *k*_*s*_ are the longitudinal wave vectors for pump, idler and signal fields, respectively, and *L* is the length of the nonlinear medium^27,28^. Therefore, in standard crystals the phase matching condition *Δk* = *0* needs to be satisfied in order to have efficient emission, which implies well defined directions for signal and idler photons. However, in 2D materials the vanishing thickness ensures that *ΔkL~0*, regardless of *Δk*, and only the transverse momentum needs to be conserved. Therefore the relaxation of (longitudinal) phase matching conditions in a deeply subwavelength medium leads to new signatures of SPDC, which strongly differ from the well-known SPDC generation from a bulk nonlinear crystal: (1) the spectrum of the SPDC is only defined by the energy conservation, regardless of the momentum, which implies a broader spectrum; (2) in contrast to a bulk nonlinear crystal, where the efficiency of the SPDC depends on the pitch & yaw angle of the crystal with respect to the pump, in the subwavelength thickness of a nonlinear crystal this angle dependence vanishes; (3) in absence of (longitudinal) phase matching conditions, the generation of photon pairs can be over a 4π solid angle, including the possibility of back-to-back photon emission^29^; (4) The SPDC emission is not polarised along a preferential direction (see Supplementary).

Besides the fundamental interest in quantum optics, there are strong technological reasons to find a 2D material with optical nonlinearity strong enough to allow for the amplification of quantum vacuum. The idea of using a type-I optical parametric oscillator (OPO) with a highly nonlinear medium of subwavelength thickness was first proposed to study analogues of the Dynamical Casimir Effect^30^. Such a nearly 2D system embedded into a cavity could obviously have important implications for integrated quantum cavity electro-dynamics and generation of squeezed light from one very compact device. Very recently emission of photon pairs via Spontaneous Four-Wave Mixing (SFWM) in a 100 nm thin layer of carbon nano-tubes has been demonstrated^31^. There is another very recently published paper which presents a very strong SHG from bulk (124 nm thickness) 3 R MoS_2_ which also has broken symmetry structure^32^. A benefit of using the multilayer crystal is the lower PL signal in comparison to the monolayer crystal. Using bulk crystal makes it easier to use grown samples with larger area which is a very important advantage for both experimental and future practical applications. Measuring higher ${\chi }^{(2)}$ and lower PL intensity in addition to having a larger area of the crystal make this type very promising for future study of the SPDC process from subwavelength materials. Although not “deeply subwavelength”, the last recently mentioned works are an important step towards dynamical Casimir-like emission from nonlinear thin films.

In this paper, we present the first theoretical and experimental assessment of the potential of exfoliated MoS_2_ monolayers as a 2D source of SPDC. In the visible range, we accurately measure the spectral dependence of the ${\chi }^{(2)}$ and design an experiment to test the SPDC emission when the material is pumped at 436 nm, corresponding to the ${\chi }^{(2)}$ maximum. The cross-correlation data are dominated by the residual, strong PL signal and, due to the limitations of our single photon counting electronic device and collection efficiency, no significant quantum correlations could be observed. However, our estimates show that, despite the strong PL background, the measurement of single photon emission could be within experimental reach with slightly better electronics and improved collection efficiency. In order to remove the PL signal, we then proceed to the IR region, by pumping at 780 nm, where the ${\chi }^{(2)}$ is still close to the maximum value, and studying the emission at 1560 nm. Here we present polarisation and life-time data, which are in agreement with our theoretical model, providing indications of the presence of SPDC emission from MoS_2_ monolayer. No significant cross-correlation data could be acquired in this spectral region, due to the extremely low photon count rate. We also discover and characterise a relatively strong IR emission from the edges of multi-layer MoS_2_ and clearly demonstrates that it qualitatively differs from the monolayer signal.

By addressing the main experimental challenges of a SPDC experiment involving a 2D material like MoS_2_, we believe that our work shows clear indications that the detection of quantum emission from MoS_2_ (or similar) monolayer is within the experimental reach in the next future, paving the way for new exciting experiments in quantum and nonlinear optics.

## Results

**Figure 1:**
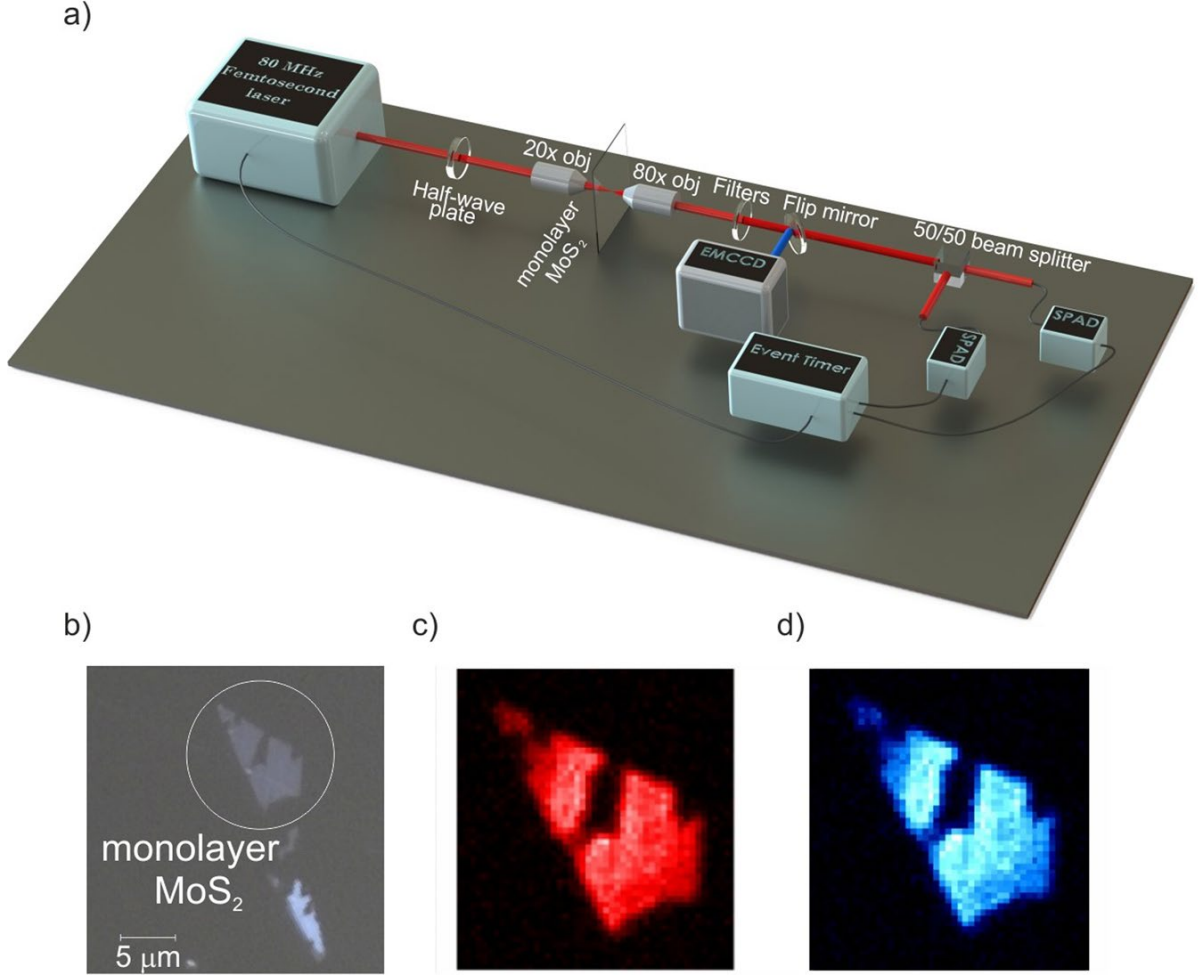
(**a**) Schematic experimental setup, (**b**) optical microscope image of mechanically exfoliated crystals (**c**) photoluminescence signal from monolayer MoS_2_ measured with bandpass filter centred around 700 nm and (**d**) second Harmonic Generation (SHG) from the same monolayer crystal measured with a band-pass filter centred around 393 nm.

Figure 1(a) shows a schematic of our setup. In Fig. 1(c,d) we show an EMCCD image of the PL signal from a MoS_2_ monolayer pumped at *400* *nm* and an image of the second harmonic generation signal from the same crystal pumped at 780 nm. A comparison of Fig. 1(c,d) show similar uniform photon generation from the surface of the monolayer MoS_2_ for PL and SHG, suggesting that there is a unique physical mechanism behind the enhancement of PL and second order nonlinear susceptibility ${\chi }^{(2)}$.

**Figure 2:**
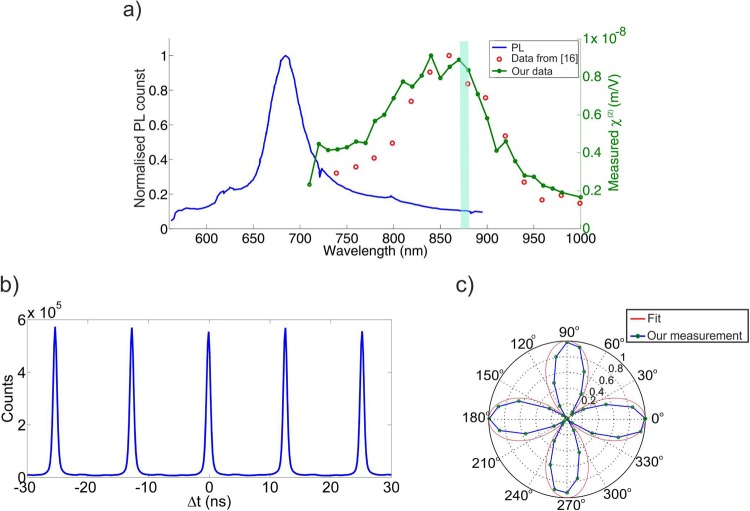
(**a**) Photoluminescence signal and measured ${\chi }^{(2)}$ from monolayer MoS_2_: the blue line is the measured spectrum of the photoluminescence signal (under 437 nm excitation); the red circles are ${\chi }^{(2)}$ values measured in literature^16^ (not in scale, magnified 20 times); the green line with dots represent our experimental data for ${\chi }^{(2)}$ measurement, where the right hand side y-axis corresponds to the real calculated value of ${\chi }^{(2)}$ in our experiment, (**b**) Cross-correlation measurement with *50 nm* bandwidth centred around *875 nm*, (**c**) SHG polarisation dependence measurement with a pump wavelength at *875 nm*.

First, we aim to characterise ${\chi }^{(2)}$ as a function of the fundamental wavelength in the range between *710 nm (1*.*75 eV)* and *1000 nm (1*.*24 eV)*. Figure 2(a) compares our results with reference^16^. The shape of the spectrum is similar and we also present a better resolution, but we find a maximum value of ${\chi }^{(2)}={10}^{4}pm/V$, whereas the values from^16^ are about 20 times smaller (*400 pm/V* at the maximum). However, our values are one order of magnitude less than those reported by^15^ and thus overall compatible with the range of values reported in literature.

In Fig. 2(a) we also plot the measured PL signal from a monolayer under *437 nm* excitation. This shows that the maximum of ${\chi }^{(2)}$ around *875 nm* should correspond to a relatively low level of PL emission. In Fig. 2(c) we report a polarisation characterisation of the SHG, which is a useful technique to investigate the crystal symmetry of films and probe the relative grain orientation. In order to take this measurement, we rotate the polarisation of the pump beam with the sample fixed and a polariser with fixed angle in front of the EMCCD camera. A sixfold symmetry in the SHG polarization dependence is expected for normal incidence on *D*_*3h*_ crystal symmetry, when an analyser is set parallel (or perpendicular) to the pump direction and the crystal is rotated around the normal direction, as in^15^. Here we use a slightly different technique, which is more suitable to our setup, we keep the sample fixed, set a polariser before the detectors and rotate the polarisation of the pump beam through a half wave plate (HWP). With this method, a fourfold pattern should be expected, as clearly described by^33^ (see Supplementary) and indeed this is what we observe in Fig. 2(c).

We then investigate SPDC using a BBO crystal to generate the second harmonic of *875 nm* pulses (*437 nm*) and use this beam to pump the MoS_2_ monolayer. A beam of *5 µm* in diameter is used to excite a region of the monolayer where the SHG signal is particularly high. We pump the crystal with *18 µW* pump power and measure 37000 counts/s on each detector.

We perform cross-correlation measurement on the signal as explained in methods. Figure 2(b) shows the cross-correlation measurement of the signal from the MoS_2_ monolayer over two hours of integration time using a filter with *50 nm* bandwidth centred around *875 nm*. As expected, most of the observed counts are due to the PL signal, as confirmed by lifetime and polarization measurements (data not shown), and therefore the measured coincidence-to-accidental ratio (CAR) is about 1. However, in order to see if this data is compatible with an eventual SPDC emission we evaluate the expected number of coincidences N_expected_ due to quantum emission and compare this number with the standard deviation of the peak maxima σ_experimental_, which is about 27000 counts (see Supplementary). Our estimation for the number of detectable photons in the visible regime, which is about 1930 proves that the measurable number of generated entangled photon pairs is much less (about 14 times) than the experimental standard deviation in the cross-correlation measurement. This experimental standard deviation is about 15 times higher than the theoretical standard deviation.

The result of cross-correlation measurement in visible range is not surprising as in the visible range the PL signal is quite strong and the SPDC signal is dominated by it. Therefore, we move on to a study of SPDC signal in the IR regime, where we do not expect a significant PL signal. Probing a wavelength as far in the IR as possible, is likely to eliminate any residual PL. However, by pumping at the wavelength where ${\chi }^{(2)}$ is maximum (875 nm) would result in an emission centred around 1750 nm, where the efficiency of IR (InGaAs/InP) single photon detectors is very low. By also taking into account the quantum efficiency of the detectors we choose to pump at *780 nm*, where ${\chi }^{(2)}$ is still approximately 60% of the maximum value, but the corresponding SPDC signal lies around the wavelength of detectors’ maximum efficiency (QE ~ 10%). Since we expect a broad emission, we use a 80 nm pass band filter centred around 1560 nm to probe the emission, in order to collect a large signal.

**Figure 3:**
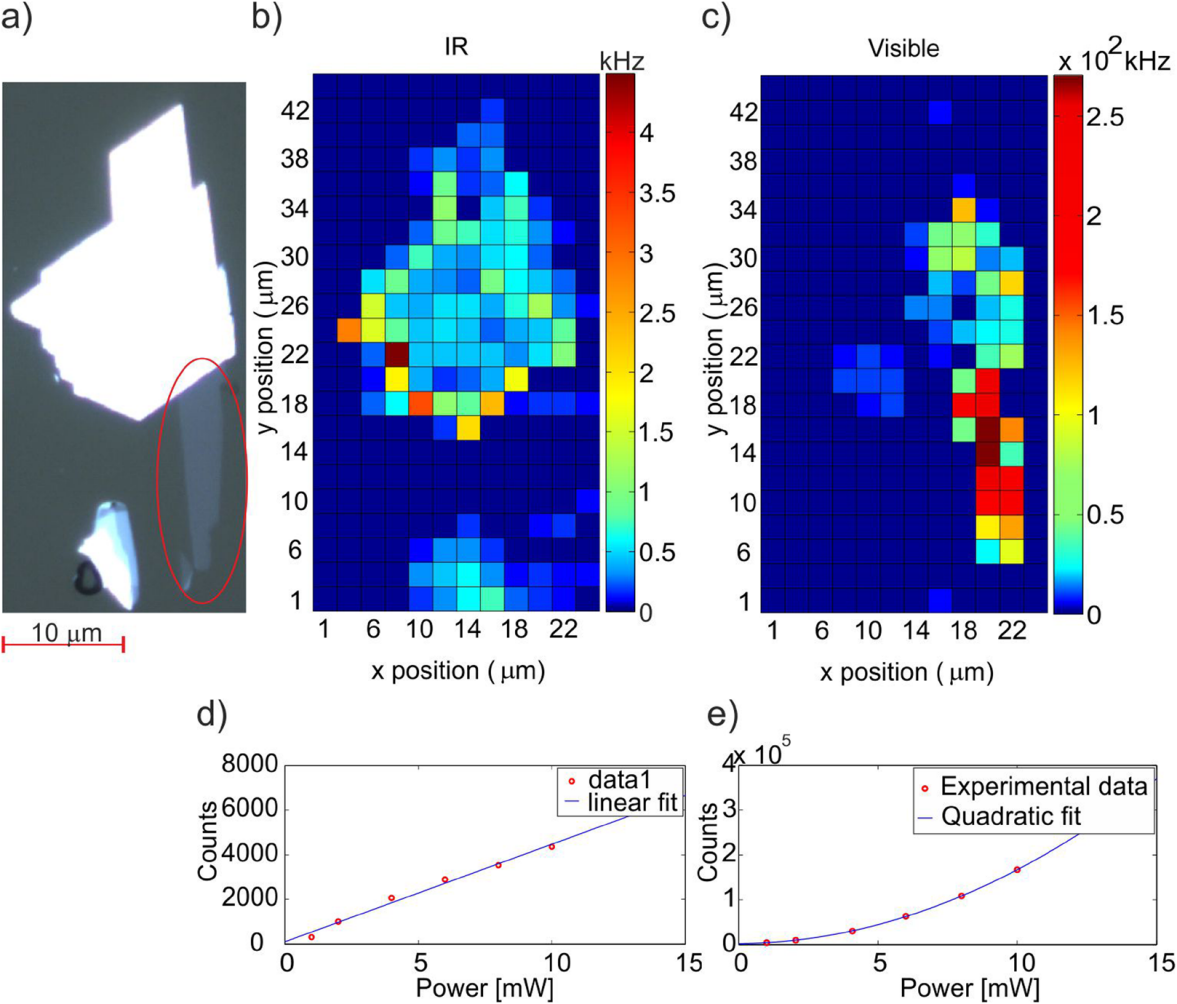
(**a**) Microscope image of the crystal. The monolayer region is indicated by the red oval, (**b**) IR signal intensity (counts) from the monolayer and bulk crystal, (**c**) SHG signal intensity (counts) from a monolayer and a bulk crystal of MoS_2_ with *2 µm* resolution. The pump wavelength for both SHG measurement in the visible and measurement of IR signal is *780 nm*, (**d**) Pump-power dependence of the IR signal, (**e**) Pump-power dependence of the SHG signal.

As a first step, we compile a map of the IR signal from a typical MoS_2_ sample consisting of a monolayer flake attached to a ‘bulk’ (several layers) crystal, as shown in the optical image in Fig. 3(a).

Figure 3(b) shows that a relatively strong IR signal is detected all over the bulk part of the crystal, in particular around the edges, and is quite intense in particular points. The monolayer and the few layers of crystal on the bottom show a one-two orders of magnitude lower emission intensity. Figure 3(c) shows for comparison the topography of the SHG signal obtained with a *780 nm* pump, with a large signal coming from the monolayer and some bright spots around the right edge, which likely corresponds to small single layer flakes. Finally, in Fig. 3(d,e) we present a power dependence for the IR and SHG signals. SHG presents the typical quadratic dependence on the pump power, whereas the IR signal at 1*560 nm* is roughly linear. A linear dependence is compatible with SPDC but also with any kind of PL emission.

**Figure 4:**
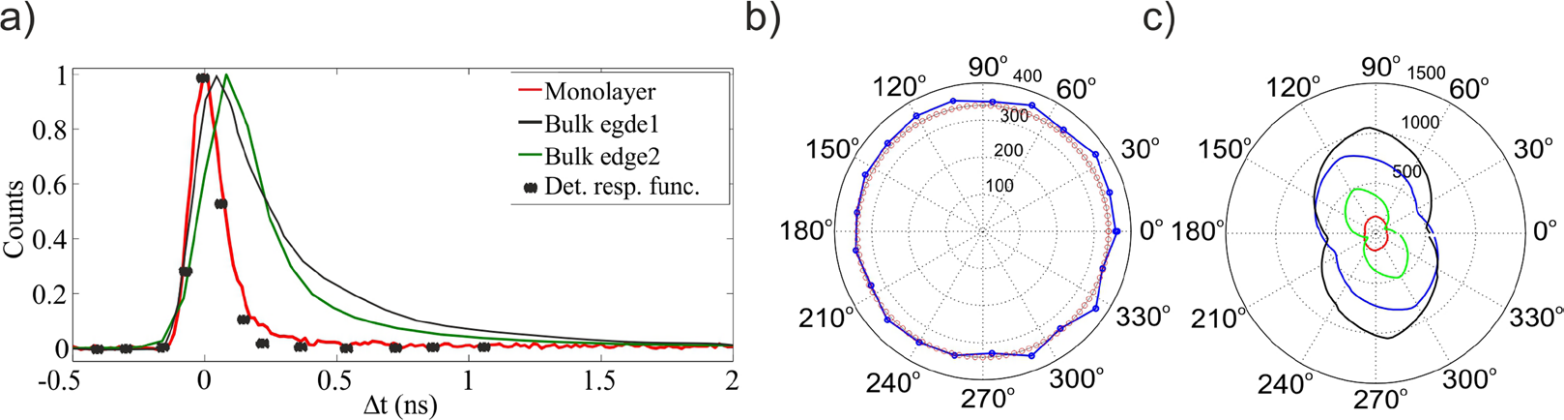
(**a**) Time-resolved IR signal from the mechanically exfoliated MoS_2_ crystal. The red curve represents the life-time of IR signal centred around *1560 nm* from the monolayer crystal, which corresponds to the response function of the SPAD (black dots). The black and green curves represent the counts found for two different points of bulk crystal’s edge. The Black dots represent the response function of the detector, (**b**) Measurement of the polarisation of the IR signal centred around *1560 nm* from the monolayer MoS_2_. The red circles show the theoretical calculation results (see Supplementary), (**c**) Pump polarisation dependence and polarisation of generated IR signal centred around *1560 nm* from bulk edge of MoS_2_ crystal.

In order to investigate the origin of the IR signal both from edges and from MoS_2_ monolayers we perform polarization and life-time measurement as illustrated in Fig. 4.

In Fig. 4(a) we show the lifetime properties of the IR signal by time-correlated single photon counting, using a trigger pulse from the fs laser. Different points on the bulk edges give different lifetimes, as exemplified by the black and green curves. When fitted these curves (see Supplementary for details) always reveal two exponential contributions (${\rm{counts}}(t)=A{e}^{-t/{\tau }_{1}}+B{e}^{-t/{\tau }_{2}}$, with *A*, *B τ*_1_ and *τ*_2_ being fitting parameters), with roughly the same decay times of *τ*_*1*_ = *0.15 ns* and *τ*_*2*_ = *4*.*7 ns* and different weights from point to point on the crystal. We verify that the fast component corresponds to the response function of the detectors and the electronics, thus indicating a lifetime shorter than 0.15 ns for this process.

Strikingly the signal from the monolayer only presents the fast component (see Supplementary Information for details), as shown by the red line in Fig. 4(a). The slower decay *(4*.*7 ns)* observed from multilayer regions (black curve) is indicative of some form of PL. The “instantaneous” response (with respect to the response function of the measurement system, represented by the black points in Fig. 4(a)) is compatible with SPDC emission, which is expected to be nearly instantaneous^28^. In Fig. 4(b) we present the polarization measurement results for the IR emission of a single monolayer. In this case no pump or signal polarisation dependence is observed. The absence of polarisation dependence is compatible with a SPDC model (see Supplementary for details). In Fig. 4(c) we present the polarisation dependence of both the pump and of the generated IR signal centred around *156*0* nm* from an area on the bulk edge of MoS_2_ crystal. In the first case, we rotate the half wave-plate before the focusing objective from *0* to *180°* and keep the polariser before the SPADs fixed at an angle which maximises the signal (black curve) and at *90°* from there (red curve). In the second case, we rotate the polariser from *0* to *360°* and fix the polarisation of the pump beam to maximise the signal (blue curve) and at *45°* from there (green curve). In both cases, the polar plot displays a two-lobed behaviour, which is an indication of a linear polarisation dependence. Different points on the bulk edge of the MoS_2_ crystal show different behaviours in terms of brightness, visibility and phase of signal polarisation. Some areas have little or no dependence on the pump polarisation but the IR signal is strongly polarized. Other areas show the opposite behaviour. A sample of this varied scenario is illustrated in the Supplementary. Since the polarisation and lifetime of the signals emitted from the monolayer and the bulk edge states have very different features, we infer that they have likely different physical explanations. The polarisation dependence of the emitted IR signal from the monolayer is fully compatible with our expectations from the theoretical SPDC model. Furthermore, the short lifetime of the IR emission from the monolayer, which corresponds to the response function of the detector indicates the instantaneous generation of the signal, which is the typical behaviour for SPDC signal, while the lifetime of IR emission from the bulk and edge state and its polarisation dependence are qualitatively different. These signs lead us to believe that the observed IR signal from the monolayer belongs to the SPDC process, while the IR signal from the bulk and edge states are due to PL emission. However, no significant cross-correlation data could be acquired in this spectral region, due to the extremely low photon count rate.

## Methods

### All our measurements are performed at room temperature

In Fig. 1(a) we present the experimental setup. We use a 110-fs Ti-Sapphire oscillator with 80-MHz repetition rate and tuneable wavelength from 680 to 1000 nm. The laser polarisation is controlled by a half wave plate (HWP). Additionally, a BBO crystal can be used to up-convert the light to the UV-visible range. The laser beam is then focused into the sample by a 20×, 0.4 NA objective (O1) to a spot which can be tuned between a few and tens of micrometres in diameter for the data shown in Fig. 3. The sample is mechanically exfoliated from bulk MoS_2_ and transferred on to a 500 µm thick fused silica substrate. The light generated by pumping the MoS_2_ crystals is then collected by a high numerical aperture objective 80×, 0.85 NA (O2) and imaged by an Andor EMCCD camera or sent into single photon detectors (visible: Excelitas, IR: IDQuantique), after spectral filtering (F) to remove the pump beam. A linear polariser (P) is added in order to characterise the emission. Two single photon detectors (D1 and D2) are placed after a 50/50 non-polarising beam splitter in order to measure photon correlations. The signals from the detectors feed into a time to digital converter for time-correlated single photon counting (TDC, ID801 from IDQuantique), which also receives a trigger pulse from the laser, used for lifetime measurements.

We use this setup to measure the wavelength dependence of ${\chi }^{(2)}$, which is presented in Fig. 2(a). A single MoS_2_ monolayer is pumped with a focused beam with a diameter much larger than the crystal dimension so that the intensity can be considered uniform over the excited area. The SHG is collected from the second objective in a transmission configuration^34^ and imaged into an EMCCD after a short pass filter with cut-off at 550 nm. In order to calculate the value of the second order nonlinear susceptibility (${\chi }^{(2)}$) of exfoliated monolayer MoS_2_, we use a formula which relates the intensity of SHG to the pump intensity *I*_*ω*_^15^:

1 $${I}_{2\omega }=\frac{1}{8}(\frac{{\omega }^{2}.{d}^{2}}{{n}_{2\omega }.{n}_{\omega }^{2}.{c}^{3}.{\varepsilon }_{0}}).{|{\chi }^{(2)}|}^{2}.{I}_{\omega }^{2}\cdot $$

where n_2ω_ ≈ 4.5, n_ω_ ≈ 6^35^, ω is the pump frequency and d = 0.65 nm is the thickness of the monolayer.

We use the same setup in Fig. 1 for the cross-correlation measurement. To perform the cross-correlation measurement of the emitted signal from MoS_2_ monolayer in visible regime, we use a BBO crystal to generate the second harmonic of 875 nm pulses (437 nm) and use this beam to pump the MoS_2_ monolayer and use a filter with 50 nm bandwidth centred around 875 nm.

The main assumption for the calculation of measurable photon pairs from SPDC in MoS_2_ monolayers is the pure isotropic emission, i.e. each photon of a pair can be emitted in any possible direction due to the lack of longitudinal momentum conservation, while the total transverse momentum is still conserved, due to the lack of longitudinal momentum conservation. This strongly limits the maximal achievable CAR due to the limited collection efficiency.

## Electronic supplementary material

Supplementary information

## Data Availability

All relevant data present in this publication can be accessed at 10.17861/38a16cef-1543-4397-848a-c6030338f484.
