# Author Correction: Towards spontaneous parametric down conversion from monolayer MoS_2_

**DOI:** 10.1038/s41598-019-43801-7

**Published:** 2019-05-21

**Authors:** Hatef Dinparasti Saleh, Stefano Vezzoli, Lucia Caspani, Artur Branny, Santosh Kumar, Brian D. Gerardot, Daniele Faccio

**Affiliations:** 1 Institute of Photonics and Quantum Sciences, SUPA, Heriot-Watt University, Edinburgh, EH14 4AS United Kingdom; 2 Institute of Photonics, Department of Physics, University of Strathclyde, Glasgow, G1 1RD United Kingdom

Correction to: *Scientific Reports* 10.1038/s41598-018-22270-4, published online 01 March 2018

The original version of this Article omitted an affiliation for Lucia Caspani. The correct affiliations for Lucia Caspani are listed below:

Institute of Photonics and Quantum Sciences, SUPA, Heriot-Watt University, Edinburgh, EH14 4AS, United Kingdom.

Institute of Photonics, Department of Physics, University of Strathclyde, Glasgow, G1 1RD, United Kingdom.

This has now been corrected in the HTML and PDF versions of this Article, and in the accompanying Supplemental Material.

