# Supplementary material for: Towards spontaneous parametric down conversion from monolayer MoS2
Source: Sci Rep. 2018 Mar 1;8:3862. doi: 10.1038/s41598-018-22270-4 (PMC5832751; doi:10.1038/s41598-018-22270-4)
Supplement: Supplementary file 1 — Supplementary information [file 41598_2018_22270_MOESM1_ESM.docx]

**Towards spontaneous parametric down conversion from monolayer MoS_2_**

**Hatef Dinparasti Saleh^1^, Stefano Vezzoli^1^, Lucia Caspani^1,2^, Artur Branny^1^, Santosh Kumar^1^, Brian D. Gerardot^1^, and Daniele Faccio^1*^**

**^1^**Institute of Photonics and Quantum Sciences, SUPA, Heriot-Watt University, Edinburgh EH14 4AS, United Kingdom

^2^Institute of Photonics, Department of Physics, University of Strathclyde, Glasgow G1 1RD, United Kingdom

*d.faccio@hw.ac.uk

Supplementary

Estimation of the SPDC contribution for the MoS_2_ cross-correlation measurement

In Figure 2 (b) we present the cross-correlation measurement of monolayer MoS_2_, while pumping the crystal at 437 nm and measuring at 875 nm with a bandwidth of 50 nm. As one can see, the CAR for this measurement is around 1. We assume that the main reason for the lack of CAR>1 in this case is that only a very small part of the collected signal is due to SPDC process and this is dominated by the strong PL signal at this wavelength. In order to estimate the number of photon pairs, which will be counted in the zero-delay peak in the cross-correlation, we use the following formula ^1^ :

where, *d_eff_* is the effective nonlinear susceptibility, *P_p_* is the pump power, *L* the crystal length, *δλ_s_* is the bandwidth of signal, *λ_p_* is the pump wavelength, *λ_s_* is the signal wavelength and *λ_i_* is the idler wavelength. This formula was originally proposed to calculate the second order nonlinear susceptibility, but also to estimate parametric down conversion brightness ^2^. By using$d_{eff}=\frac{1}{2}\chi^{(2)}$^3^, we can use the value given in Figure 2 to calculate the photon flux generated by the sample for *P_p_*=18 µW and *Δλ_s_*=50 nm. Taking into account the experimental configuration, the collection efficiency and the losses we estimate the number of coincidences *N_generated_*, which should be counted in the zero-delay peak in the cross-correlation measurement. By definition, the generation of SPDC photon pairs are not phase matched in the MoS_2_ monolayer. This condition leads to the series of consequences. The most important one is the generation of the SPDC on a full solid angle 4π, which strongly affect the collection efficiency.

It can be seen that the signal power in Equation (1) scales quadratically with χ^(2)^. We should notice that the value of χ^(2)^ for MoS_2_ reported in literature varies of 3 orders of magnitude; for this estimation we use the value of χ^(2)^ that we measured at 875 nm (10^4^ pm/V, Figure 2 (a)). This value is in the middle of the reported range and we estimate that it is accurate within a factor 3, based on the experimental sources of error.

The parameters for this calculation are:

**Table 1. Input data for the estimation of SPDC brightness.**

| Variable | unit | variable | unit | variable | unit | variable | unit |
| --- | --- | --- | --- | --- | --- | --- | --- |
| $L=0.68$ | $nm$ | $P_{p}=18$ | $\mu W$ | $d_{eff}=4.5$ | $nm/\mathrm{V}$ | $n_{p}=2.2$ | $-$ |
| $\delta\lambda_{s}=50$ | $nm$ | $\lambda_{p}=437$ | $nm$ | $\lambda_{s}=875$ | $nm$ | $\lambda_{i}=875$ | $nm$ |

By inserting all these parameters into Equation (1), we get:

Then we divide the signal power by the photon energy in order to find the rate of expected SPDC photon pairs emitted from the crystal:

$$N_{generated}=\frac{\delta P_{s}\lambda_{s}}{hc}=1.4\cdot10^{5} \frac{1}{s}$$

The next step in this calculation is the estimation of the collection efficiency µ. This parameter plays a critical role in the estimation of SPDC contribution in zero-delay peak as it has quadratic contribution in the estimation. The lack of phase-matching conditions in the SPDC process for subwavelength films means that the emission is expected to be isotropic on a full solid angle of 4π, along every possible direction. Since we collect only from one side of the sample and we separate signal and idler photon non-deterministically with a 50/50 beam splitter this will contribute a factor 1/4 to *µ* and the collection efficiency of the objective can be estimated from *NA*=0.85. Then we consider collection efficiency of the transmission line, coupling efficiency into the fibers and quantum efficiency of the detectors. Taking into account all these parameters gives us a final efficiency $\mu=1.4\cdot10^{-3}.$By multiplying the rate of generated photons by *µ^2^* we find the number of detected photons as coincident photons in the cross-correlation measurement:

$$N_{generated}\mu^{2}=0.268$$

Then we multiply this number by the integration time (2 hours) of the measurement in order to find the contribution of the real detected coincident photon pairs in zero-delay peak:

$$N_{generated}\mu^{2}\Delta t=1929$$

Theoretical calculation of the standard deviation for the peak height in the measurement is about 1800, while the experimental standard deviation is 27000. This calculation shows that our experimental standard deviation is about 14 times higher than the estimated coincident photon numbers for the whole measurement, which is the reason why the CAR is basically one. Increasing the acquisition time would not improve the situation since the experimental standard deviation is dominated by electronic noise and it is far larger than the shot noise. Our estimation suggests that a viable strategy to improve the CAR consists in increasing the collection efficiency of the objective.

*Theoretical calculation of the polarisation dependence of the SHG and SPDC from MoS_2_* monolayer.

Figure 5 (a) presents a schematic view of the experimental setup for polarisation measurements. In this setup, we control the pump polarisation with a half-wave plate (HWP) and select the output polarisation by a linear polariser (Pol).

Assuming the MoS_2_ monolayer lying in the *x-y* plane and the incident pump beam traveling along the *z* axis, we define 2 systems of reference for the polarisation measurements. As illustrated in Figure 5 (b) $\theta$is the angle of the pump polarisation given by the electric field $\overrightarrow{E_{p}}$with respect to the mirror plane y and$\alpha$is the angle of the polariser with respect to the same axis.

In both SPDC and SHG the generated field is proportional to the nonlinear polarisation:

$$P_{i}(\omega_{1}+\omega_{2})=\varepsilon_{0}\sum_{ijk} \chi_{ijk}^{(2)}(\omega_{1}+\omega_{2})E_{j}(\omega_{1})E_{k}(\omega_{2}).$$

where i, j and k are indexing the directions with respect to the crystal axes. MoS_2_ monolayer is a D3h crystal, thus we have ^3^:

$$\chi_{yyy}^{(2)}=-\chi_{yxx}^{(2)}=-\chi_{xxy}^{(2)}=-\chi_{xyx}^{(2)}\equiv\chi$$

and all the other terms of the nonlinear susceptibility vanish.


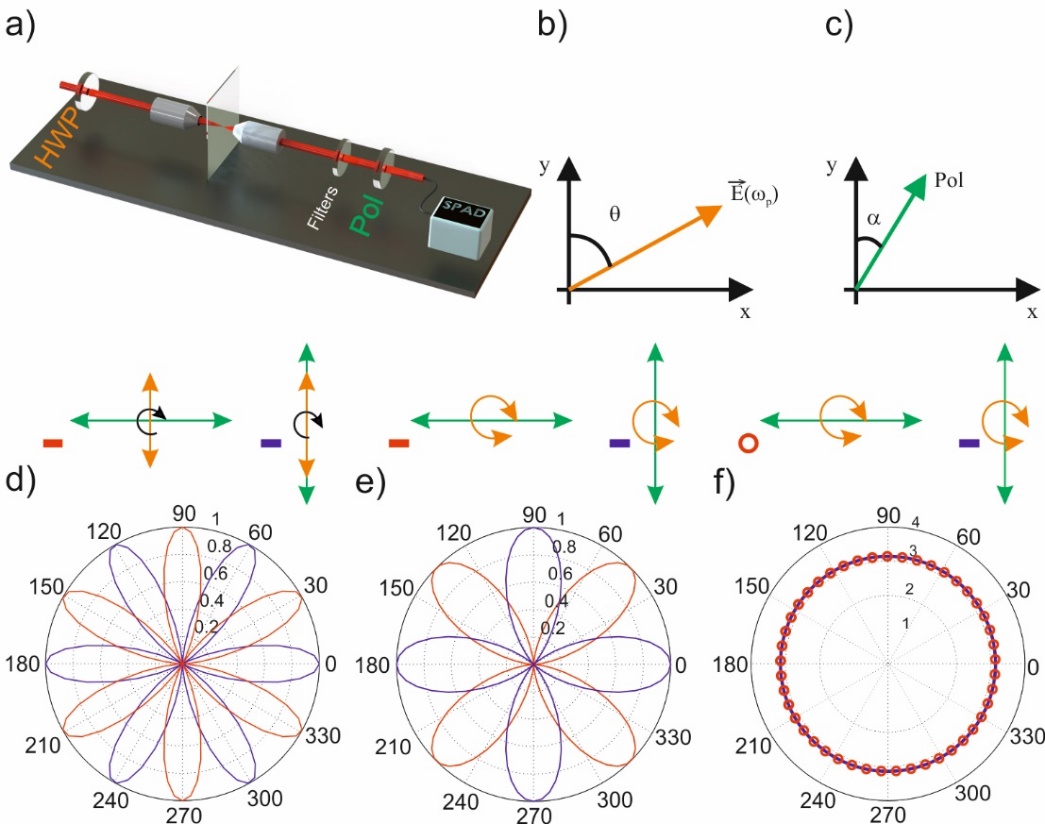


**Figure 5** (a) Experimental setup for the polarisation dependence characterisation, (b) Orientation of the pump polarisation with respect to the mirror plan, (c) Orientation of the linear polariser with respect to the mirror plane, (d) Six-folded pattern for the SHG polarisation dependence when we have the polariser angle perpendicular to the pump polarisation and rotate them together (red line) and polariser angle is parallel to the pump polarisation and rotate them together (blue line), (e) Four-folded pattern for the SHG polarisation dependence when we keep the polariser angle horizontal and rotate the pump polarisation (red line) and fixed vertical polariser axis and rotate the pump polarisation (blue line), (f) Polarisation dependence of SPDC when we keep the polariser axis fixed at any random angle *α_0_* (here *α_0_=0* – blue line – and *π/2* – red circles*)* and rotate the pump polarisation. The graphs above panels (d)-(f) represent the legend: the orange and green arrows represent the pump polarisation and polariser angle, respectively. The circular arrow indicates which angle is rotated, and the black circular arrow indicates that both the pump and polariser angles are rotated simultaneously.

In SHG a pump beam at$\omega_{p}$generates a signal at$2\omega_{p}$:

$$P_{i}(2\omega_{p})=\varepsilon_{0}\sum_{ijk} \chi_{ijk}^{(2)}(2\omega_{p})E_{j}(\omega_{p})E_{k}(\omega_{p})$$

Where:

$$\left\{\begin{matrix} E_{x}=E_{p}.\sin(\theta) \\ E_{y}=E_{p}.\cos(\theta) \end{matrix}\right.$$

By using Equation (7) and Equation (9) the 2 components of the nonlinear polarisation become:

$$\left\{\begin{matrix} P_{x}(2\omega)=-\varepsilon_{0}\chi E_{p}^{2}\sin(2\theta) \\ P_{y}(2\omega)=\varepsilon_{0}\chi E_{p}^{2}\cos(2\theta) \end{matrix}\right.$$

We can write the polarisation *P* along the polariser axis in terms of$\alpha$:

$$P_{\alpha}=P_{y}\cos(\alpha)+P_{x}\sin(\alpha)$$

In most of literature the authors fix both the pump and the polariser angles $\theta$ , $\alpha$ and rotate the sample. This is equivalent to fixing the sample and rotating both pump and analyser together, which in our notation corresponds to just setting$\alpha=\theta$(parallel, $P_{\parallel}$) or$\alpha=\theta+\frac{\pi}{2}$(perpendicular, $P_{\perp}$) in Equation (11):

$$P_{_{\parallel}}(2\omega)=\varepsilon_{0}\chi E_{p}^{2}\cos(2\theta)\cos(\theta)-\varepsilon_{0}\chi E_{p}^{2}\sin(2\theta)\sin(\theta)=-\varepsilon_{0}\chi E_{p}^{2}\cos(3\theta)$$

And similarly for $P_{\perp}$. The SHG intensity is proportional to the modulus square of *P*:

$$\left\{\begin{matrix} I_{\parallel}(2\omega)\propto\cos^{2}(3\theta) \\ I_{\perp}(2\omega)\propto\sin^{2}(3\theta) \end{matrix}\right.$$

This case gives the standard and well-known six-folded pattern and is represented in Figure 5 (d).

An alternative way to perform the same measurement is what we presented in the main text, i.e. keeping the polariser fixed at some angle $\alpha_{0}$and rotating the pump polarisation angle $\theta$. In this case the intensity of the emitted signal along $\alpha_{0}$is:

$$I_{\alpha_{0}}(2\omega)\propto(\cos(2\theta)\cos(\alpha_{0})-\sin(2\theta)\sin(\alpha_{0}))^{2}$$

In Figure 5 (e) we plot the case for $\alpha_{0}=0,\pi/2$and always find a 4-fold pattern. This is indeed what we observed in the SHG experiment.

For calculating the polarisation dependence of SPDC we just start from considering a seed at $\omega_{s}$and calculate the nonlinear polarisation along direction *i* at the idler frequency ($\omega_{id}$) from Equation (7):

$$P_{i}(\omega_{id}=\omega_{p}-\omega_{s})=\varepsilon_{0}\sum_{jk} \chi_{ijk}(\omega_{p}-\omega_{s})E_{j}(\omega_{p})E_{k}(-\omega_{s})$$

where$\omega_{p}$, $\omega_{s}$ and$\omega_{id}=\omega_{p}-\omega_{s}$ are the pump, signal and idler frequencies, respectively. Since in SPDC$\overrightarrow{E_{s}}$is only given by vacuum fluctuations we have to average the result on all possible directions of the polarisation vector$\overrightarrow{E_{s}}$.

Assuming the$\overrightarrow{E_{s}}$at a certain angle$\theta_{s}$ with respect to y, Equation (10) becomes:

$$\left\{\begin{matrix} P_{x}(\omega_{p}-\omega_{s})\propto\{\sin(\theta)\cos(\theta_{s})-\cos(\theta)\sin(\theta_{s})\} \\ P_{y}(\omega_{p}-\omega_{s})\propto\{\cos(\theta)\cos(\theta_{s})-\sin(\theta)\sin(\theta_{s})\} \end{matrix}\right.$$

Again if we consider a polariser at a fixed angel$\alpha_{0}$we have:

$$P_{\alpha_{0}}(\omega_{p}-\omega_{s})=P_{x}(\omega_{p}-\omega_{s})\sin(\alpha_{0})+P_{y}(\omega_{p}-\omega_{s})\cos(\alpha_{0})$$

The intensity is proportional to the square of the polarisation:

$$\begin{matrix} I_{\alpha_{0}}(\omega_{p}-\omega_{s})\propto[\sin(\alpha)(\sin(\theta)\cos(\theta_{s})+\cos(\theta)\sin(\theta_{s}))+ \\ +\cos(\alpha)(\cos(\theta)\cos(\theta_{s})-\sin(\theta)\sin(\theta_{s}))]^{2} \end{matrix}$$

In the spontaneous amplification case, we have to consider a seed given by the vacuum fluctuations at all the $\theta_{s}$, so that:

$$\begin{matrix} I_{_{\alpha_{0}}}^{SPDC}(\omega_{p}-\omega_{s})\propto\int_{0}^{2\pi} d\theta_{s}\{\sin(\alpha_{0})[\sin(\theta)\cos(\theta_{s})+\cos(\theta)\sin(\theta_{s})]+ \\ +\cos(\alpha_{0})[\cos(\theta)\cos(\theta_{s})-\sin(\theta)\sin(\theta_{s})]\}^{2}=\pi\end{matrix}$$

Therefore, as illustrated in Figure 5 (f) for any choice of the polariser angle $\alpha_{0}$ no dependence of the SPDC signal on the pump polarisation angle is expected, as indeed observed in our measurements (Figure 4 (b)).

On the contrary, the IR signal measured on the bulk crystal presents a different polarisation signature, as illustrated in the main text. In Figure 6 we are presenting a few supplementary measurements realised in different points of the sample. This behaviour has been characterised by both rotating the polarisation of the pump, as described before, but also by rotating the angle $\alpha$ of the polariser, while fixing the pump polarisation, as sketched in Figure 6.


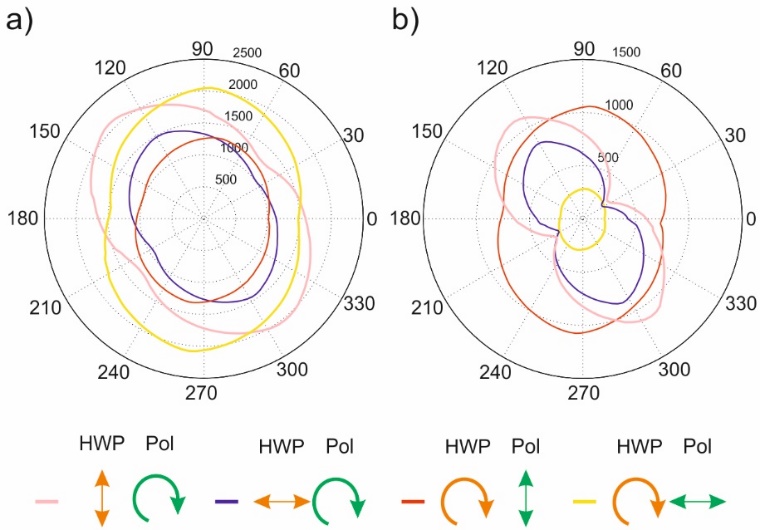


**Figure 6**. (a) Polarisation dependence and polarization of IR signal (centred around 1560 nm) from bulk region and (b) bulk edge of the same crystal.

Measurement of sample thickness

Figure 7 shows the sample height measurement of the crystal shown in Figure 1 (b). We use Atomic Force Microscopy method to be sure about the number of layers of the crystal. Notwithstanding that the visible contrast inspection is a confident method for us to make sure about the number of layers, we use AFM inspection as an additional check.


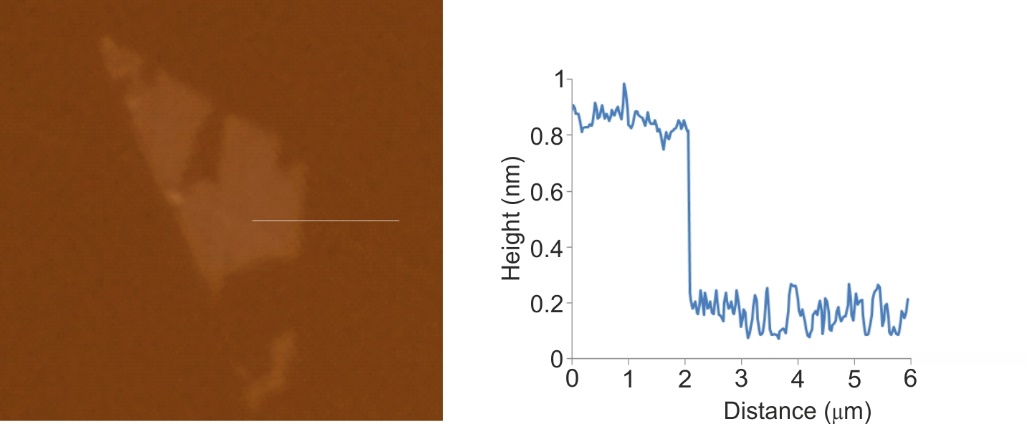


**Figure 7**. Atomic force microscopy measurement of the sample from Figure 1(b).

Life-time decay fit

Figure 8 presents the fit for the red and black curves (presented in Figure 4(a)), which correspond to the signal from monolayer and bulk edge crystals, respectively.


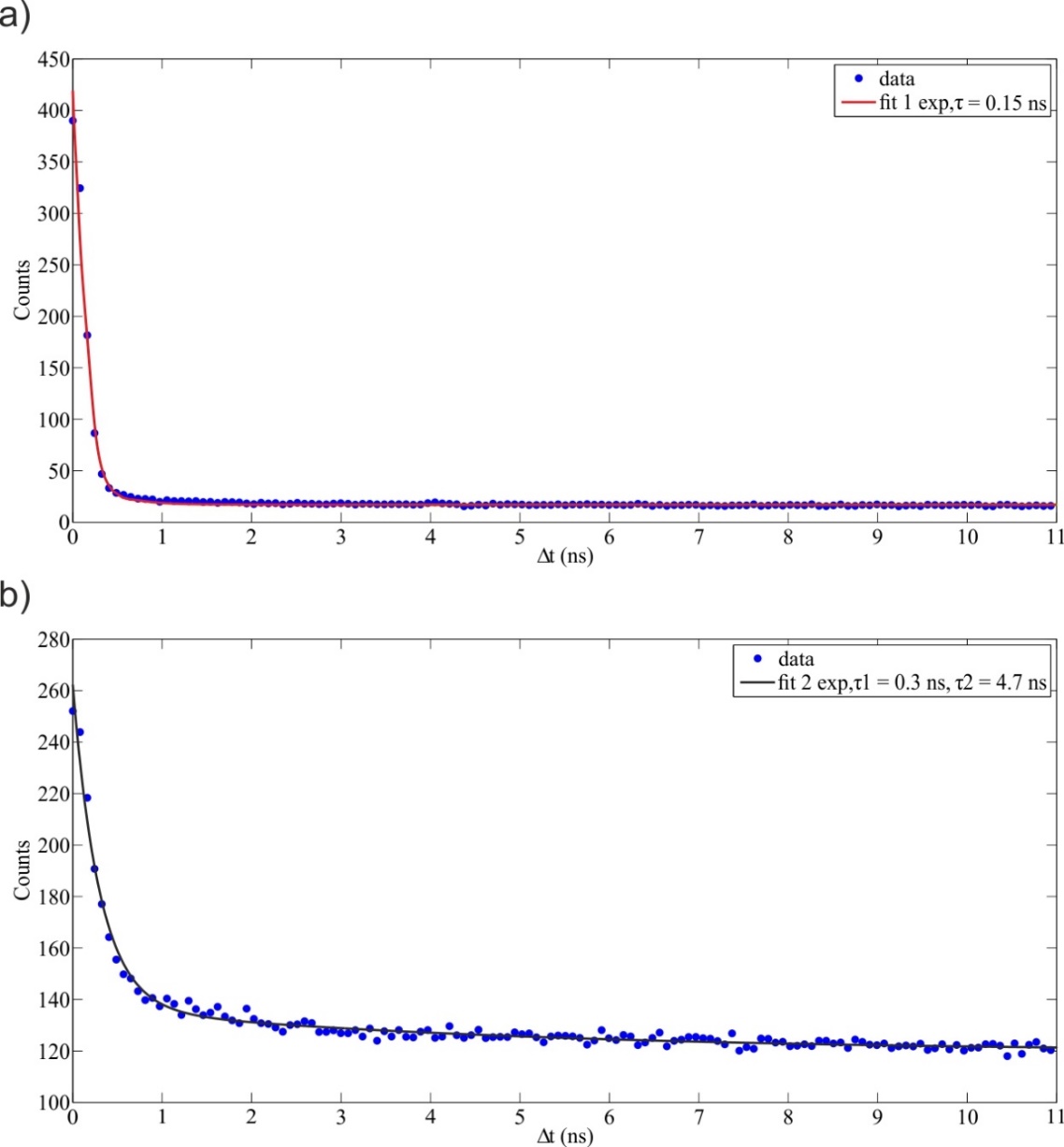


**Figure 8**. Fit of the IR emitted signals presented in Figure 4 (a). (a) Fit for the time-resolved IR signal from MoS_2_ monolayer, (b) fit for the time-resolved emitted IR signal from the bulk edge.

**References**

1. Brida, G., Genovese, M. & Novero, C. On the measurement of photon flux in parametric down-conversion. *Eur. Phys. J. D* **8,** 273–275 (2000).

2. Cheung, E. C., Liu, J. M., Koch, K. & Moore, G. T. Measurements of second-order nonlinear optical coefficients from the spectral brightness of parametric fluorescence. *Opt. Lett.* **19,** 168–170 (1994).

3. Boyd, R. *Nonlinear Optics*. (Elsevier Wordmark, 2008).
